# GPX7 Is Targeted by miR-29b and GPX7 Knockdown Enhances Ferroptosis Induced by Erastin in Glioma

**DOI:** 10.3389/fonc.2021.802124

**Published:** 2022-01-20

**Authors:** Yan Zhou, Haiyang Wu, Fanchen Wang, Lixia Xu, Yan Yan, Xiaoguang Tong, Hua Yan

**Affiliations:** ^1^ Clinical College of Neurology, Neurosurgery and Neurorehabilitation, Tianjin Medical University, Tianjin, China; ^2^ Tianjin Key Laboratory of Cerebral Vascular and Neurodegenerative Diseases, Tianjin Neurosurgical Institute, Tianjin Huanhu Hospital, Tianjin, China; ^3^ Clinical Laboratory, Tianjin Huanhu Hospital, Tianjin, China

**Keywords:** bioinformatics, ferroptosis, glioma, GPX7, miR-29b

## Abstract

**Background:**

Glioma is a lethal primary tumor of central nervous system. Ferroptosis is a newly identified form of necrotic cell death. Triggering ferroptosis has shown potential to eliminate aggressive tumors. GPX7, a member of glutathione peroxidase family (GPXs), has been described to participate in oxidative stress and tumorigenesis. However, the biological functions of GPX7 in glioma are still unknown.

**Methods:**

Bioinformatics method was used to assess the prognostic role of GPX7 in glioma. CCK8, wound healing, transwell and cell apoptosis assays were performed to explore the functions of GPX7 in glioma cells. *In vivo* experiment was also conducted to confirm *in vitro* findings. Ferroptosis-related assays were carried out to investigate the association between GPX7 and ferroptosis in glioma.

**Results:**

GPX7 was aberrantly expressed in glioma and higher expression of GPX7 was correlated with adverse outcomes. GPX7 silencing enhanced ferroptosis-related oxidative stress in glioma cells and the loss of GXP7 sensitized glioma to ferroptosis induced by erastin. Furthermore, we found that miR-29b directly suppressed GPX7 expression post-transcriptionally. Reconstitution of miR-29b enhanced erastin sensitivity, partly *via* GPX7 suppression.

**Conclusions:**

Our study clarified the prognostic role of GPX7 in glioma and preliminarily revealed the role of GPX7 in ferroptosis, which may be conducive to the exploration of therapeutic targets of glioma.

## Introduction

Glioma is the most common and lethal tumor of the central nervous system, and glioblastoma (GBM, grade IV) is the most malignant subtype (5-year survival are only about 5.5%) (1). Considering the high recurrence and mortality rates of glioma, it is crucial to investigate its causes and potential molecular mechanisms to find new targets for early diagnosis and personalized treatment.

To defend oxidative stress, the living organisms have evolved several antioxidative mechanisms that prevent cells from damage induced by reactive oxygen species (ROS) (2). Among these mechanisms, the glutathione peroxidase (GPXs) family is a major antioxidant enzyme family (GPX1-GPX8) that reduces various ROS such as hydrogen peroxide and lipid peroxides (3).

In the past, GPX7 was viewed as an important intracellular sensor that detects redox level and transmits ROS signals to multiple biologic processes (4). Recent studies have reported that GPX7 participated in the initiation and progression of tumors. DunFa Peng et al. (5) reported that GPX7 had tumor suppression function in oesophageal adenocarcinomas and was silenced by promoter DNA methylation. Zheng Chen et al. (6) showed that GPX7 was downregulated in gastric cancer and reconstitution of GPX7 suppressed tumor growth in 3D organotypic models. E. Guerriero et al. (7) showed that GPX7 had an overexpression in hepatocellular carcinoma tissues. In glioma, only one bioinformatic study reported that GPX7 was a potential prognostic molecule based on Chinese Glioma Genome Atlas (CGGA) database (8). However, the mechanisms by which GPX7 might exert its actions in the development of glioma remain to be elucidated.

Ferroptosis is a newly discovered type of necrotic cell death caused by the accumulation of lipid-based ROS and has gained growing interest on account of its close relevance to multiple pathological situations (e.g. neurodegeneration, ischemia/reperfusion injuries and malignancies) (9, 10). Several small molecule compounds have been developed to trigger ferroptosis of tumors. Erastin is the most commonly used ferroptosis inducer, which directly suppresses the cystine/glutamate antiporter (system x_c_^-^) to suppress GSH synthesis (11, 12). GPX4, known as the key enzymatic inhibitor of ferroptosis, can protect biological membranes from peroxidative degradation (10). Yang et al. reported that the inhibition of GPX4 elevated the lipid peroxidation level and induced the ferroptosis of tumors (13). However, we need to take into account the dependence of lipid metabolism and the abundance of GPX4 in the specific tissues. Pharmacological targeting of GPX4 may only achieve partial anti-tumor effects (14). Based on the antioxidative functions and complex roles of GPX7 in tumors, we speculate that GPX7 may participate in glioma development by regulating ferroptosis, which need to be further verified.

MicroRNAs (miRNAs) are endogenous small RNA molecules, which inhibit gene expression by binding directly to complementary sequences located mostly in the 3′-untranslated regions (3′UTR) of mRNAs (15). In cancers, miRNAs can elicit notable effects on cell phenotypes by suppressing the expression of target genes (16). However, to date, whether GPX7 expression is regulated by miRNA has not been reported.

Our study, for the first time, disclosed that GPX7 was targeted by miR-29b and we preliminarily explored the relationship between GPX7 and ferroptosis. Integrating bioinformatic analysis and experimental analysis allow more effective contributions to the promising target of GPX7 in glioma.

## Materials and Methods

### Dataset Selection

The transcript level of GPX7 in glioma was assessed by Oncomine, The Cancer Genome Atlas (TCGA) (17), GSE16011 dataset (from Gene Expression Ominibus database) (18) and the Repository of Molecular Brain Neoplasia Data (REMBRANDT) (19). Apart from that, the protein level of GPX7 was validated by 127 glioma tissue samples obtained from Huanhu Hospital (Department of Neurosurgery, Huanhu Hospital, Tianjin, China; 2017). All cases were primary gliomas and patients received no extra treatments such as radiotherapy and chemotherapy before surgery. All the biopsies were acquired from surgical resection and processed immediately into formalin-fixed, paraffin-embedded tissues to produce the tissue array. The clinicopathological features of patients were concluded in **Table S2**.

### Cells and Chemicals

The human glioblastoma cell lines U87, T98G and LN229 were purchased from Beijing Beina Chuanglian Biotechnology Institute and A172 and T98G were purchased from iCell Bioscience Inc. Co. Ltd. (Shanghai, China). Erastin (T1765), RSL3 (T3646), ferrostatin-1 (fer-1, T6500), liproxstatin-1 (lip-1, T2376) and deferoxamine (DFO, T1637) were purchased from TOPSCIENCE (Shanghai, China).

### Cell Transfection and Lentivirus Infection

The sense oligonucleotide sequences of siRNAs and miRNAs mimics were concluded in **Supplementary Table S1**. The transfection of siRNA and miRNA mimics was conducted using Lipofectamine 2000 (Thermo Fisher Scientific, MA, USA). The establishment of stable GPX7 knockdown cell line *via* lentivirus vectors (LV3-shGPX7, the sequence was shown in **Table S1**) was conducted by GenePharma (Suzhou, Jiangsu, China). The siRNAs, miRNA mimics, knockdown lentivirus vectors and pcDNA3.1-GPX7 overexpression plasmid were purchased from GenePharma (Suzhou, Jiangsu, China).

### Quantitative Real-Time PCR (qPCR)

The qPCR was performed according to the processes, as previously reported (20).The primers were designed as follows: GPX7 forward, 5′-AGTAGCCCCAGATGGAAAG-3′ and reverse, 5′-TCGCTTCAGTAGGATGAGC-3′; GAPDH forward, 5′-CAATGACCCCTTCATTGACC-3′ and reverse, 5′-GACAAGCTTCCCGTTCTCAG-3′. Relative quantification was analyzed by the 2^−ΔΔ^Ct method.

### Western Blot and Immunohistochemistry (IHC)

Western blot assay was carried out according to the manufacturer’s protocol, as previously described (20). The blocked membrane was incubated with antibody against GPX7 (1:500; 13501-1-AP, Proteintech, IL, USA) or β-actin (1:1000; CST, MA, USA) for at least 4 hours. After incubation with second antibody for 60 min, chemiluminescence and exposure were performed.

Immunohistochemistry assay was completed by two experienced pathologists from Pathology Department of Huanhu Hospital. Patients were grouped into high and low expression cohorts grounded on the integrated scores of GPX7 expression, as previously reported (21). The primary antibodies used in IHC were Ki67 (ab16667, abcam, UK), MMP2 (D4M2N, CST, USA), N-cadherin (D4R1H, CST, USA).

### CCK-8, Wound Healing, Invasion and Apoptosis Analysis

Cells were seeded in 96-well plates (5 × 10^3^ cells per well) and transfected with siGPX7 or co-transfection of miR-29 mimics and pcDNA3.1-GPX7 plasmid. CCK-8 agent (K009-500; ZETA LIFE, CA, USA) was injected to each well for 30-minute incubation at 0, 24, 48 and 72h time points, respectively. Finally, microplate reader (iD5, Molecular Devices, CA, USA) was employed to measure the absorbance at 450 nm. For wound healing assay, cells were cultured in 12-well plates. Next, the bottoms of wells were scratched with a pipette tip. After being washed twice, cells were cultured within the serum-free medium for 24h. Finally, the wound closure (%) was measured by ImageJ. Transwell and apoptosis assays were performed according to the manufacturer’s protocols, as previously described (22).

### Glutathione Assay

GSH Detection Kit (Solarbio Co., Beijing, China) was used to detect reduced glutathione (GSH) according to the manufacturer’s instructions.

### Lipid Peroxidation Assay

The level of lipid peroxidation was detected using BODIPY 581/591 C11 (GLPBIO, CA, USA) according to the manufacturer’s instructions.

### Cellular Iron Concentration Assay

The iron assay kit (FerroOrange, F374, Dojindo Molecular Technology) was used to detect cellular iron concentration level according to the manufacturer’s protocols.

### Dual Luciferase Reporter Assay

The wild/mutant-type 3′UTR of GPX7 was inserted into the GP-miRGLO vector (Promega, WI, USA). LN229 and T98G cells were cotransfected with wild or mutant vectors and NC mimic or miR-29a/b/c-3p mimic. The activities of luciferase were measured 48h after transfection according to the manuals of the Dual Luciferase Assay System (Promega, WI, USA).

### Immunofluorescence Assay

Cells plated onto poly-L-lysine-coated glass coverslips were fixed with 4% paraformaldehyde for 20 min. The cells were permeabilized with 0.5% Triton X-100 (Sigma, USA) and blocked by 5% BSA for 2 h. The coverslips were incubated with primary antibody and secondary antibody following the manufacturer’s protocol. Primary antibodys were GPX7 (1:500; 13501-1-AP, Proteintech, IL, USA), Ki67 (1:1000, ab16667, abcam, UK), MMP2 (1:800, D4M2N, CST, USA) and N-cadherin (1:1000, D4R1H, CST, USA). Secondary antibody was Alexa Fluor^®^ 488-conjugated Anti-Rabbit IgG (H+L) (1:800, Jackson ImmunoResearch Inc, USA). After being mounted with antifade mounting medium with DAPI (ZSGB-BIO, Beijing, China), images were acquired under a confocal microscope (LSM 800, Zeiss, Germany)

### Xenograft Model With Nude Mice

BALB/c-A nude mice (female, 4 weeks old) were purchased from Sibeifu Beijing Biotechnology Co. Ltd. (Beijing, China). Animal assays were conducted under the approval of the Animal Care and Used Committee of Tianjin Huanhu Hospital. First, the mice were randomly allocated into four groups and 1 × 10^7^ LN229 cells stably transfected with lentiv-GPX7 or lentiv-NC were inoculated s.c. into the axillary fossa of mice every two groups. When tumor volume reached approximately 50 mm^3^, one of every two groups were treated with erastin dissolved in 5% DMSO/corn oil (23) (30 mg/kg intraperitoneal injection, every other day) for 3 weeks. The tumor size was estimated using formula (length × width^2^/2). 42 days later, the mice were sacrificed, and the tumors were removed for IHC assay.

### Bioinformatics Analysis

The differential expression of GPXs was analyzed using GEPIA, a web database containing abundant normal specimens from GTEx database (24). Gene set enrichment analysis (GSEA) (25) was conducted with the false discovery rate (FDR) < 0.25 and normal P value < 0.05 as thresholds. The correlation of GPX7 and immune infiltration in glioma was analyzed in TIMER (https://cistrome.shinyapps.io/timer/) (26, 27), an online web portal which can investigate immune cell infiltration levels using data from TCGA.

For ferroptosis analysis, ferroptosis gene set (contributed by WikiPathways) was downloaded from Molecular Signatures Database (28). Ferroptosis and GO and KEGG gene sets related to redox biology and glutathione metabolism were analysed by GSEA. A well-established model of ferroptosis potential index (FPI) was defined as the enrichment score (ES) of positive components minus that of negative components, which was calculated using single sample gene set enrichment analysis (ssGSEA). The details of FPI model could be obtained from a previous paper (29).

### Statistical Analysis

The relationships between GPX7 protein expression and clinical variables of glioma were estimated using *Chi-Square* test. Patients with missing information were excluded from the corresponding analysis. Student’s t-test and one‐way analysis of variance (ANOVA) were used to test for significant differences between two or multiple groups, respectively. All tests were two-sided. The statistical analysis was performed using R software v3.6.3. and GraphPad Prism software.

## Results

### Preparation of Datasets

The clinical characterastics of patients from 4 datasets were concluded in **Table 1**. The detailed information of patients in Huanhu cohort were recorded in **Table S2**.

**Table 1:** The information of patients in 4 datasets.

Characteristic	TCGA	GSE16011	REMBRANDT	Huanhu
**Total**	703	284	472	127
**Age**				
≥52	263	133		45
<52	407	143		82
**Gender**				
Male	386	184	221	77
Female	284	92	126	50
**Grade**				
I		8	2	
II	248	24	98	39
III	261	85	85	35
IV	161	159	130	53
**Histology**				
Pilocytic astrocytoma		8		
Astrocytoma	192	29	147	28
Oligodendroglioma	190	52	67	46
Oligoastrocytoma	128	28		
Glioblastoma	160	159	219	53
Mixed glioma			11	
**IDH1 mutation**				
Yes	91	81		87
No	34	140		40
**1p19q codeletion**				
Yes		110		48
No		45		29
**KPS**				
<80	71	82		
≥80	341	182		

### GPX7 mRNA Levels in Different Databases

**Figure 1:**
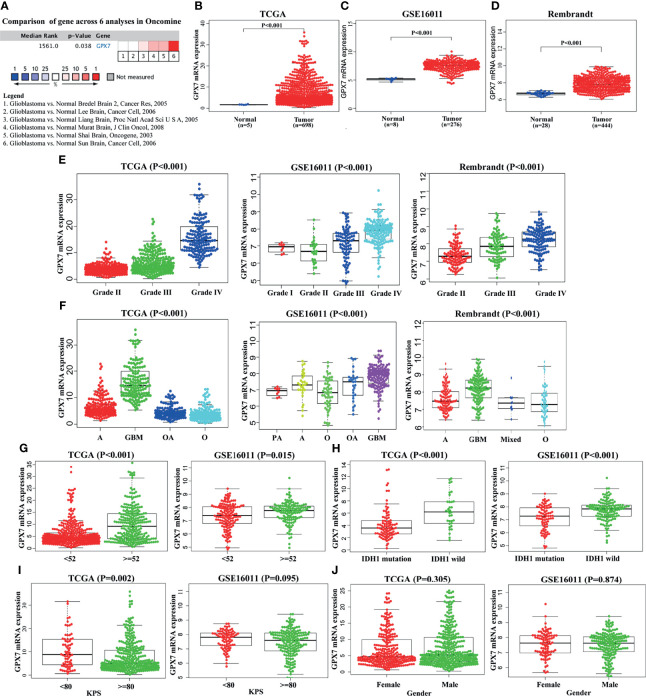
The expression of GPX7 among databases. **(A)** The expression differences of GPX7 between normal and GBM were analyzed across the six analyses in Oncomine database. **(B–D)** The expression differences of GPX7 between normal and GBM in TCGA, GSE16011 and Rembrandt datasets. **(E–J)** The relationships between GPX7 and clinical characteristics among different datasets.

Firstly, the mRNA expression level of GPX7 in GBM was analyzed in Oncomine database. Pooled analysis of six datasets revealed an upregulation of GPX7 in GBM than in normal (**Figure 1A**, P = 0.038). This result was also validated in other three datasets (**Figures 1B–D**, P < 0.001).

### GPX7 Is Related to Clinicopathological Factors of Glioma

In TCGA, GSE16011 and Rembrandt, GPX7 expression was upregulated in tumor with grade IV compared with those with lower grades (**Figure 1E**). For histopathologic type, higher expression of GPX7 was found in the patients with adverse histopathologic type (**Figure 1F**). Besides, GPX7 expression increased among older patients and those with wild-type IDH1 (**Figures 1G, H**). Moreover, some other clinicopathological factors (Karnofsky Performance Status (KPS) and gender) were also analyzed (**Figures 1I, J**). These data indicated that GPX7 high expression predicted adverse malignant phenotypes of glioma.

### GPX7 Predicts Worse Survival in Glioma

**Figure 2:**
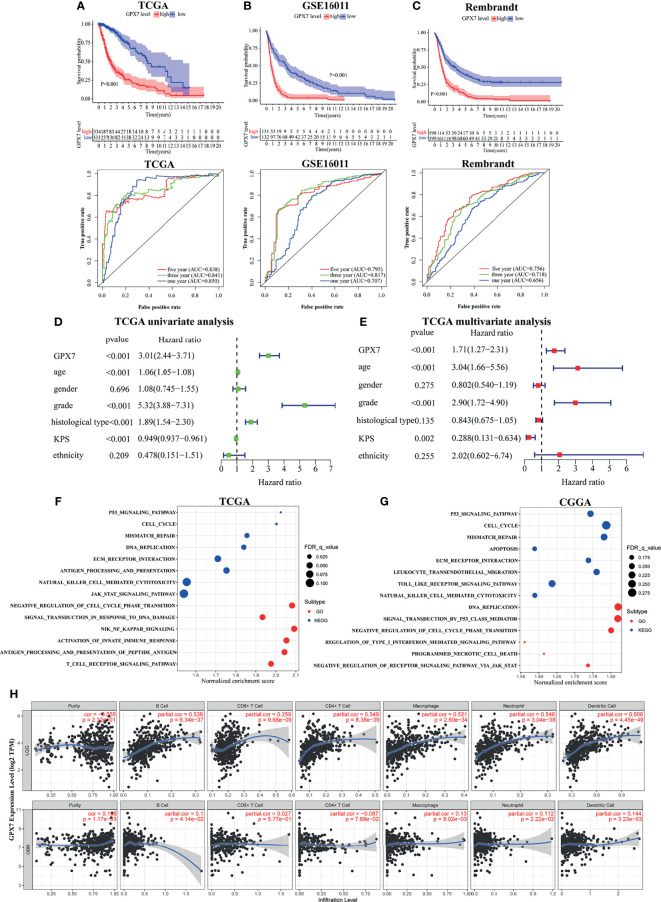
The prognostic values of GPX7 in public databases and GSEA. **(A–C)** Kaplan–Meier analysis and 1, 3 and 5- year ROC curves of survival. **(D, E)** Univariate and multivariate Cox regression in TCGA. **(F, G)** GO and KEGG enrichment analyses using GSEA method in TCGA and CGGA. **(H)** The correlation between GPX7 and immune infiltration in LGG and GBM using the TIMER database.

Patients were grouped into two cohorts based on median expression value. In TCGA, Kaplan-Meier survival analyses showed that glioma patients with GPX7-high had a worse prognosis than that with GPX7-low (**Figure 2A**). And ROC analyses demonstrated that GPX7 was a good predictor of survival (**Figure 2A**). Similar results were also obtained in GSE16011 and Rembrandt datasets (**Figures 2B, C**).

To further explore the independent prognostic value of GPX7, the univariate analysis revealed that GPX7-high correlated significantly with a worse OS (**Figure 2D**). In multivariate analysis, GPX7 was still independently correlated with OS (**Figure 2E**), along with age, grade and KPS. Additionally, we also analyzed the expression levels of other GPX family members in glioma based on GEPIA. We found GPX1, GPX3, GPX4 and GPX8 had relative high expression levels in both LGG and GBM, while GPX2 had low expression level (**Supplementary Figure S1A**). However, in multivariate analyses, none of them was independently correlated with OS (**Supplementary Figure S1B**).

### GPX7 Is Associated With Tumor and Immune Related Pathways

GSEA was employed to find the biological functions of GPX7 in glioma. In TCGA, we found some gene sets related to tumorigenesis (e.g. P53 signaling pathway and cell cycle) and immunity (e.g. natural killer cell mediated cytotoxicity and antigen processing and presentation) were enriched in KEGG analysis in the cohorts with GPX7 high expression (**Figure 2F**). For GO terms, tumorigenesis and immunity related gene sets (e.g. negative regulation of cell cycle phase transition, activation of innate immune response and T cell receptor signaling pathway) were also enriched (**Figure 2F**). Meanwhile, similar consequences were also gained in CGGA (**Figure 2G**).

To better understand the roles of GPX7 in the immune microenvironment of glioma, we analyzed the correlations between GPX7 and several common immune cell types in TIMER. As shown in **Figure 2H**, strong positive correlations existed between GPX7 expression and the infiltrations of all six immune cells types in LGG. Meanwhile, GPX7 was also positively correlated with the infiltrations of B cells, neutrophils, macrophages and dendritic cells in GBM.

### Protein Expression of GPX7 in Glioma Tissues

**Figure 3:**
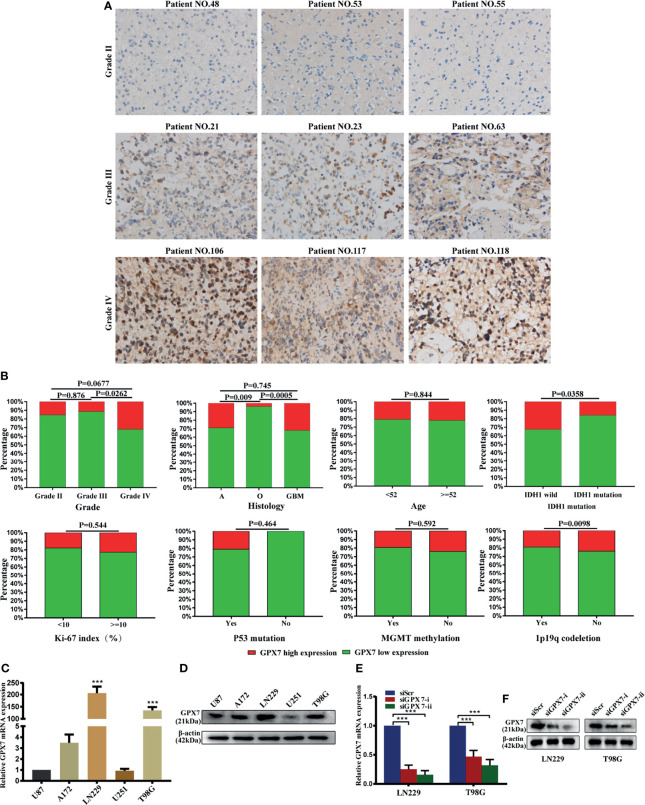
The protein expression of GPX7 in Huanhu cohort. **(A)** Representative IHC slides of specimens with grade II, III and IV. Patients’ numbers were recorded in **Table S2**. **(B)** Associations between GPX7 protein level and clinical variables in the Huanhu cohort. **(C, D)** The mRNA and protein levels of GPX7 in five different GBM cell lines using qPCR (with U87 as a control) and western blot. **(E, F)** PCR and western blot were performed to determine the expression of GPX7 after transfection with two siRNAs (siGPX7-i and siGPX7-ii). ***P < 0.001.

Representative IHC slides of specimens from Huanhu cohort with different grades are shown in **Figure 3A**. Some examples of high expression of GPX7 in patients with grade II and low expression in grade III were showed in **Supplementary Figure S1C**. We found that GPX7 had relative high expression levels in samples with grade IV, histologic GBM, IDH1 wild and in samples without 1p19q codeletion, while no association was found between GPX7 and age, Ki67, P53 mutation and MGMT methylation (**Figure 3B** and **Table S2**).

We also detect GPX7 expression in five GBM cell lines using western blot and RT-PCR methods (**Figures 3C, D**). LN229 and T98G were selected for subsequent experiments. Two siRNAs (siGPX7-i and siGPX7-ii) were used to suppress GPX7 expression (**Figures 3E, F**).

### GPX7 May be Relevant to Ferroptosis in Glioma

**Figure 4:**
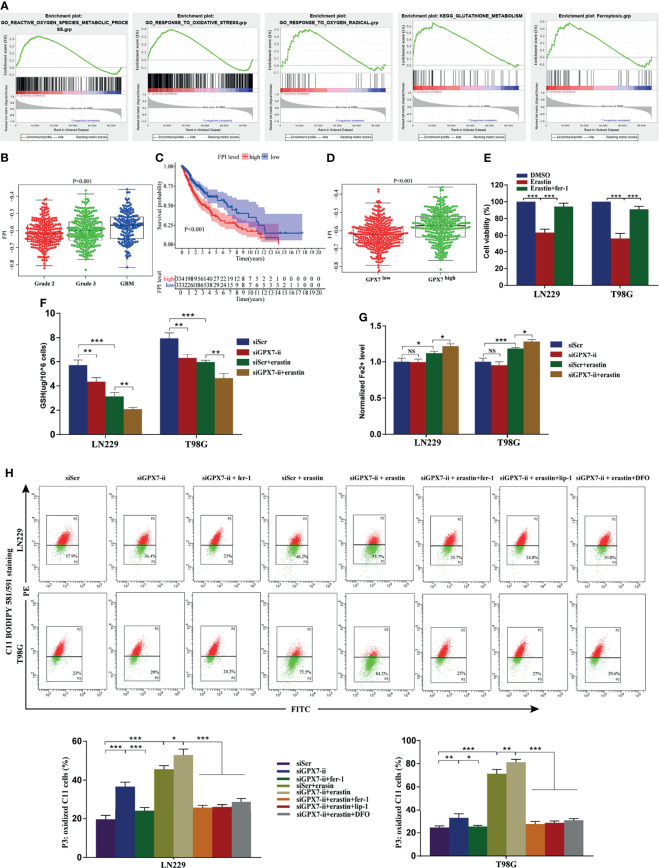
The association of GPX7 and ferroptosis in glioma. **(A–D)** Bioinformatics analysis of the association of GPX7 and ferroptosis based on data from TCGA. **(A)** GSEA of ferroptosis and redox biology related gene sets in cohort with GPX7 high and low. **(B)** The different FPI levels among different WHO grades of glioma. **(C)** Kaplan-Meier analysis of OS according to the FPI level. **(D)** The association of GPX7 expression and FPI level in TCGA glioma samples. **(E)** CCK-8 assay was used to detect the cell viability of glioma cells treated with erastin (10 μM) for 24 h with or without fer-1 (2 μM). **(F)** The reduced GSH level in glioma cells subjected to siGPX7-ii transfection with or without erastin (10 μM) treatment. **(G)** The Fe2+ concentration was measured by FerroOrange probe. **(H)** Lipid peroxidation was detected in glioma cells subjected to siGPX7-ii transfection with or without fer-1 (2 μM), erastin (10 μM), lip-1 (100 nM) and DFO (100 μM) treatment, using C11 BODIPY 581/591 probe on flow cytometry. Within each chart, cells in P3 region in green represent those stained with oxidized dye. *P < 0.05, **P < 0.01, ***P < 0.001. ns, not significant.

Given that the loss of GPX7 causes the increase of the intracellular ROS concentration and sensitizes cells to excessive environmental oxygen (30), we explored preliminarily the association between GPX7 and ferroptosis in both TCGA and CGGA. As shown in **Figure 4A** and **Supplementary Figure S2A**, GSEA revealed that ROS metabolic process, response to oxidative stress, response to oxygen radical, glutathione metabolism and ferroptosis gene sets were enriched in cohort with GPX7-high. Then, we employed the ferroptosis potential index (FPI) to represent the level of ferroptosis based on the mRNA data from the two databases. As shown in **Figures 4B, C** and **Supplementary Figures S2B, C**, higher FPI was correlated with advanced tumor grades and shorter survival time in glioma. Moreover, patients with GPX7-high tended to have higher FPI levels (**Figure 4D** and **Supplementary Figures S2D**). These data implied a potential association between GPX7 and ferroptosis in glioma.

### GPX7 Silencing Enhances Ferroptosis-Related Oxidative Stress in Glioma Cells

To verify the aforementioned bioinformatic analysis results, ferroptosis-related oxidative stress indicators, such as reduced GSH content, lipid peroxidation and Fe^2+^ concentration, were assessed in glioma cells. The inhibitor (fer-1) and inducer (erastin) of ferroptosis were employed in this research. Firstly, the susceptibility of glioma cells to ferroptosis was evaluated. CCK-8 assay showed that LN229 and T98G cells treated with erastin (10 μM) for 24 h had a significant decline in cell viability, which was blocked by fer-1 (**Figure 4E**). Among two siRNAs, we selected siGPX7-ii, which showed more effective inhibition of gene expression (**Figures 3E, F**), for the follow-up experiments. As shown in **Figure 4F**, GPX7 knockdown resulted in reduced GSH depletion in both LN229 and T98G cells, which was aggravated when combined with erastin treatment. The Fe^2+^ level was detected by FerroOrange regent. We found GPX7 deficiency didn’t affect iron level, but the combination of GPX7 deficiency and erastin treatment leaded to higher iron level than erastin treatment alone (**Figure 4G**). Lipid peroxidation is a key indicator of ferroptosis which can be detected by C11 BODIPY 581/591 probe. Compared with control group, lipid peroxidation increased significantly in the GPX7 knockdown group (**Figure 4H**). Fer−1 suppressed the accumulation of lipid peroxidation and GPX7 deficiency could enhance the effect of erastin (**Figure 4H**). We also used three ferroptosis inhibitors (lip-1, fer-1 and DFO) on GPX7 knockdown cells treated with erastin. We found that lip-1, fer-1 and DFO can abolish the increase of lipid peroxidation of GPX7 knockdown cells treated with erastin (**Figure 4H**). These results showed that GPX7 silencing could promote ferroptosis-related oxidative stress induced by erastin in glioma cells *in vitro*.

### GPX7 Silencing Synergizes With Erastin to Suppress Glioma Both *In Vitro* and *In Vivo*

**Figure 5:**
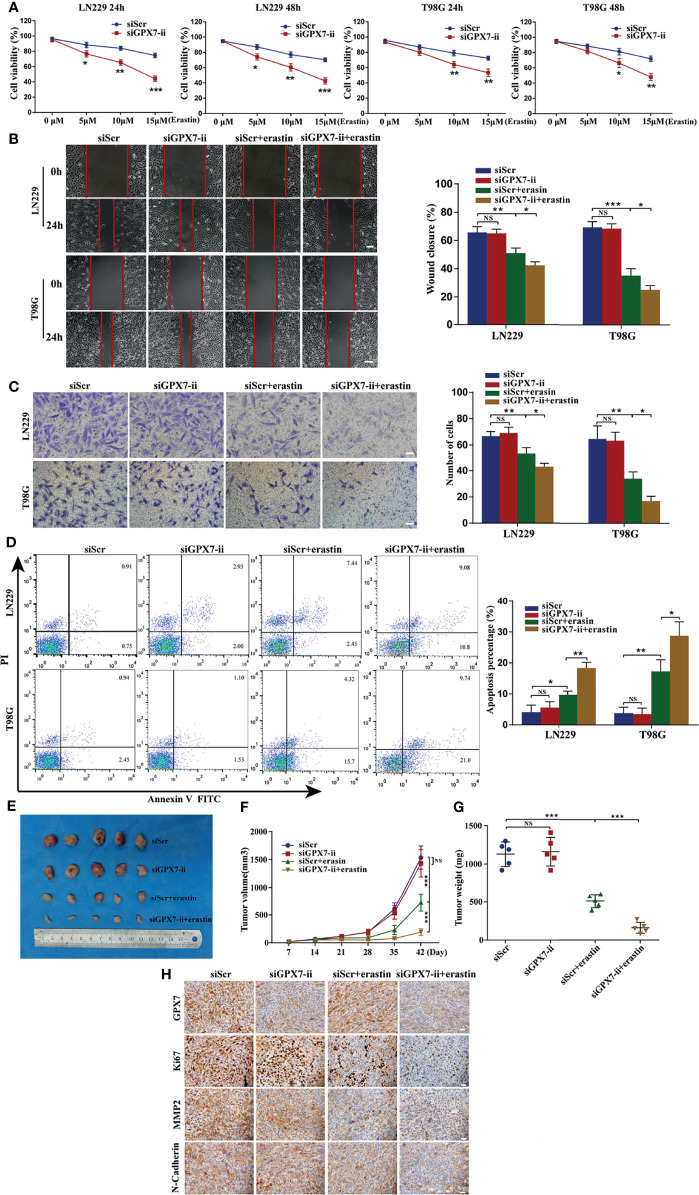
GPX7 silencing sensitizes glioma cells to erastin both *in vitro* and *in vivo*. **(A)** CCK-8 assay was applied to analyze the viability of LN229 and T98G cells treated with different concentrations of erastin following the transfection with siGPX7 and siScr. **(B)** Migration ability of cells was analyzed using wound-healing assay. Scale bar, 250 μm. **(C)** Invasive ability of cells was evaluated by transwell assay. Scale bar, 100 μm. **(D)** FITC annexin V and PI apoptosis assay. **(E)** Knockdown of GPX7 enhanced erastin-induced ferroptosis *in vivo*. The volume of tumors was shown **(F)**, and the tumor weight was measured at the endpoint **(G)**. **(H)** Immunohistochemistry staining of xenograft model-derived tumors for GPX7, Ki67, MMP2 and N-Cadherin. Scale bar = 100 μm. *P < 0.05, **P < 0.01, ***P < 0.001. ns, not significant.

Based on the regulatory effect of GPX7 on ferroptosis-related oxidative stress, we then investigated the GPX7 mediated effect on glioma development and sensitivity to erastin. CCK-8, wound healing, transwell and apoptosis flow cytometry assays were conducted to examine proliferative, migratory and invasive abilities of glioma cells with GPX7 knockdown alone or with GPX7 knockdown and erastin (10 μM) cotreatment. As shown in **Figure 5A**, CCK-8 assay revealed that GPX7 knockdown alone did not affect the proliferation of glioma cells. However, the combination of GPX7 knockdown and erastin treatment significantly suppressed the proliferation of glioma cells. This effect was more pronounced with higher erastin concentrations. In addition, GPX7 knockdown and erastin cotreatment significantly inhibited the migratory and invasive abilities (**Figures 5B, C**) and increased apoptosis (**Figure 5D**) of glioma cells, while GPX7 knockdown alone did not exhibit obvious effects. Furthermore, immunofluorescence staining revealed that the combination treatment downregulated the expressions of several indicators related to cell proliferation, migration and invasion, including Ki67, MMP2 and N-Cadherin (**Supplementary Figure S3A**). We also found that lip-1, fer-1 and DFO can suppress the apoptosis rate of GPX7 knockdown cells treated with erastin (**Supplementary Figure S3B**). Additionally, we assessed the combinational effects of GPX7 knockdown and RSL3, another ferroptosis inducing agent which inhibits directly the activity of GPX4 (13). However, the synergistic effects of GPX7 knockdown and RSL3 treatment were not obvious (**Supplementary Figures S2E, F**). These results indicated that GPX7 deficiency enhanced ferroptosis-related oxidative stress, which may not be adequate to exert obvious effects on the malignant phenotypes of glioma cells, but sensitized cells to erastin induced ferroptosis.

Then we explored whether GPX7 silencing promotes erastin-induced ferroptosis *in vivo*. A tumor xenograft model was established by subcutaneously inoculating LN229 cells infected with lentiv-NC or lentiv-GPX7, respectively. As shown in **Figures 5E–G**, the group subjected to the combination of GPX7 knockdown and erastin treatment showed a significant tumor growth inhibition, wheras the mice subjected to GPX7 knockdown alone exhibited no tumor growth suppression. Apart from that, erastin treatment alone can also suppress tumor growth. Furthermore, IHC staining revealed that the combination treatment also downregulated the expressions of Ki67, MMP2 and N-Cadherin (**Figure 5H**). These results indicated that GPX7 knockdown synergizes with erastin to inhibit glioma both *in vitro* and *in vivo*.

### GPX7 Is a Direct Target of miR-29 Family

**Figure 6:**
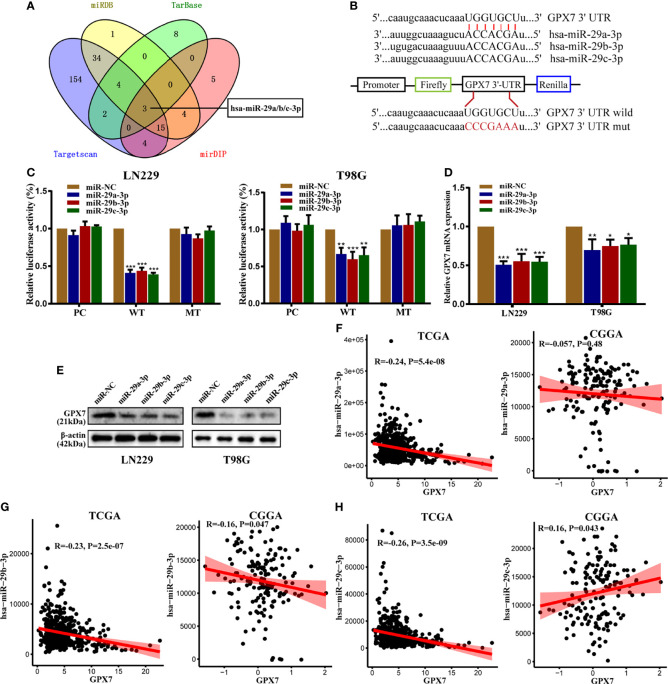
GPX7 is a direct target of miR-29 family. **(A)** The venn diagram of predicted mRNAs in four different databases. **(B)** The seed sequences of miR-29 family that match the 3′UTR of GPX7 gene and the reporter vectors containing wild-type or mutant GPX7 3’-UTR. **(C)** Dual luciferase reporter assays in LN229 and T98G, following cotransfection with miR-29 family and empty vector (pcDNA-3.1) or plasmid containing wild or mutant type 3′UTR of GPX7. **(D, E)** The mRNA and protein levels of GPX7 in cells transfected with miR-29 family mimic or miR-NC. **(F–H)** The correlations between GPX7 expression and miR-29 family in TCGA and CGGA. R: Pearson correlation coefficient. *P < 0.05, **P < 0.01, ***P < 0.001.

MiRNA is closely related to tumorigenesis, angiogenesis and chemoresistance (15). However, whether GPX7 is regulated by miRNAs is still unknown. In this research, we used TargetScan (31), miRDB (32), Tarbase (33) and mirDIP (34) databases to predict which miRNAs target GPX7. Taking the intersection of predicted results, miR-29 family (miR-29a/b/c-3p) were included (**Figure 6A**). We then used dual luciferase assays to verify our prediction. The seed sequences of miR-29 family that match the 3′UTR of the GPX7 gene were shown in **Figure 6B**. In dual luciferase assays (**Figure 6C**), transfection with miR-29a/b/c-3p mimics inhibited the WT luciferase reporter activity but did not decrease MT luciferase reporter activity. More importantly, qPCR and western blot showed that GPX7 expression was significantly impaired after elevating miR-29a/b/c expression in LN229 and T98G cells (**Figures 6D, E**). The evidence suggests that GPX7 was targeted by miR-29 family. To explore whether all miR-29 family members modulate GPX7 expression in glioma, we mined tumor gene expression profiles in TCGA and CGGA databases and found that only miR-29b-3p expression level was inversely correlated with GPX7 expression in the two databases (**Figures 6F–H**). Given that three miR-29 family members share an identical seed sequence (**Figure 6B**) and usually have similar biological functions (35), therefore, we only selected miR-29b-3p (denoted as miR-29b) for subsequent experiments.

### GPX7 Restoration Can Reverse miR-29b Mediated Enhancement of Ferroptosis-Related Oxidative Stress

**Figure 7:**
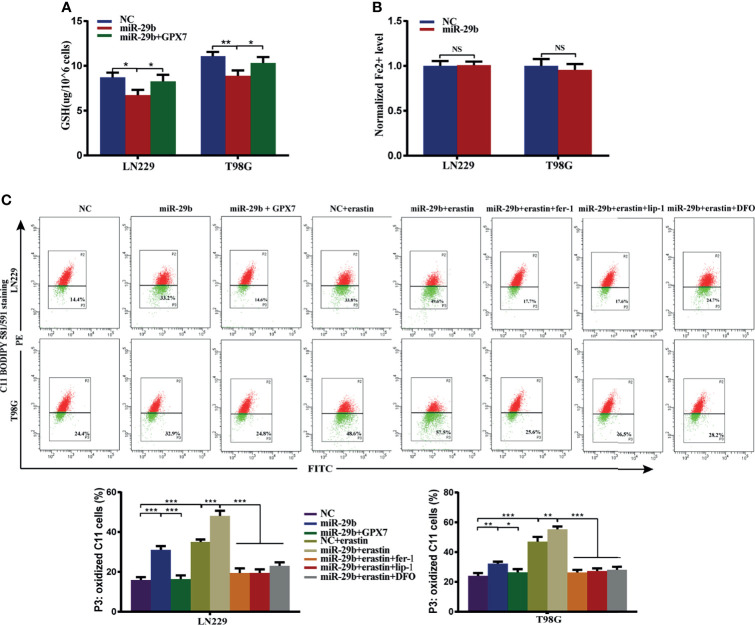
GPX7 restoration can reverse miR-29b mediated enhancement of ferroptosis-related oxidative stress. LN229 and T98G cells were transfected with miR-29b mimic or cotransfected with miR-29b mimic and GPX7 overexpression plasmid. **(A)** The reduced GSH level of cells. **(B)** The Fe2+ concentration was measured by FerroOrange probe. **(C)** Lipid peroxidation was detected in glioma cells treated with or without fer-1 (2 μM), erastin (10 μM), lip-1 (100 nM) and DFO (100 μM). Within each chart, cells in P3 region in green represent those stained with oxidized dye. *P < 0.05, **P < 0.01, ***P < 0.001. ns, not significant.

Given GPX7 being a direct target of miR-29b, we then investigated whether miR-29b also regulates ferroptosis-related oxidative stress in glioma cells. Reduced GSH, Fe^2+^ concentration and lipid peroxidation were measured. Compared with control group, GSH levels (**Figure 7A**) were markedly reduced while Fe2+ concentration wasn’t affected in the mimic group (**Figure 7B**). Furthermore, the accumulation of lipid peroxidation was significantly promoted in the mimic group (**Figure 7C**). Interestingly, we found miR-29b mediated changes in the indicators of ferroptosis were abrogated by GPX7 restoration (**Figures 7A, C**). Ferroptosis inhibitors (lip-1, fer-1 and DFO) can abolish the increase of lipid peroxidation of cells subjected to miR-29b mimic transfection and erastin treatment (**Figure 7C**). These data indicated that miR-29b can regulate ferroptosis-related oxidative stress in glioma cells, at least partially *via* GPX7.

### MiR-29b Synergizes With Erastin to Suppress Glioma Proliferation, Migration, Invasion and Induce Apoptosis Partially *via* GPX7

**Figure 8:**
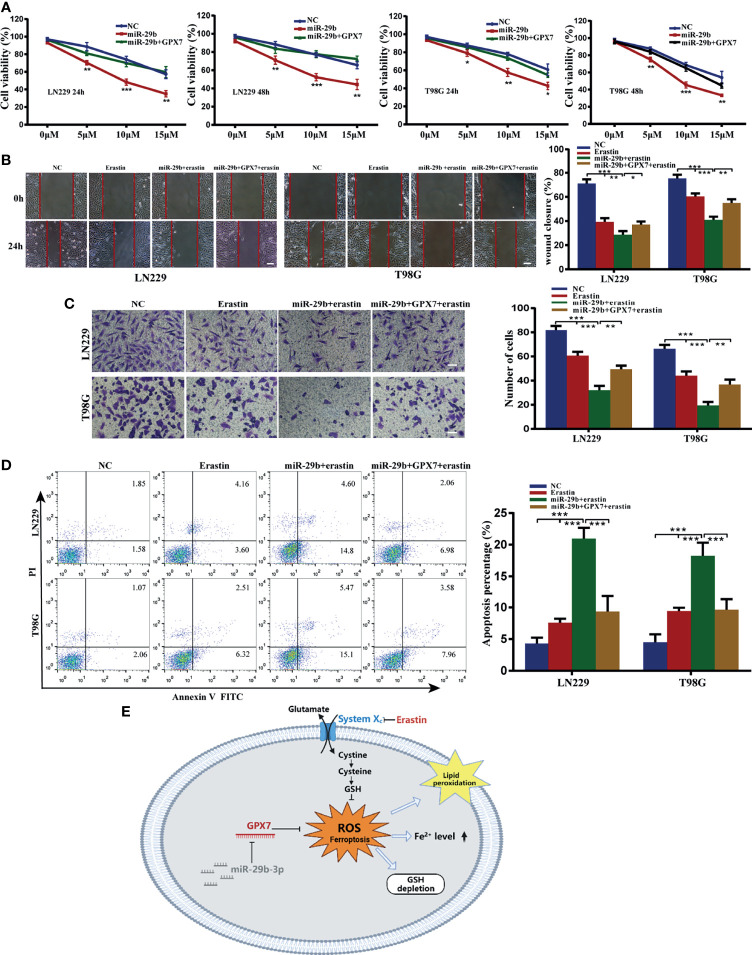
Synergistic inhibitory effects of miR-29b mimic transfection and erastin cotreatment on glioma cells, which can be partially reversed by GPX7 restoration. **(A)** LN229 and T98G cells transfected with miR-29b mimic or cotransfected with miR-29b mimic and GPX7 overexpression plasmid were treated with erastin at different concentrations. Proliferation ability of cells was evaluated using CCK-8. **(B)** Wound healing assay. The concentration of erastin is 10 µM. Scale bar, 250 μm. **(C)** Transwell assay with 10 µM erastin used. Scale bar, 100 μm. **(D)** Annexin V and PI apoptosis assay. The concentration of erastin is 10 µM. **(E)** The schematic diagram of the roles of miR-29b/GPX7 in glioma ferroptosis induced by erastin, which was created on the BioRender.com. *P < 0.05, **P < 0.01, ***P < 0.001.

The above results stimulated us to explore whether miR-29b can sensitize glioma cells to erastin. Using the same experimental assays employed above, we found that LN229 and T98G cells with miR-29b mimic transfection and erastin cotreatment attenuated proliferative (**Figure 8A**), migratory (**Figure 8B**) and invasive (**Figure 8C**) abilities and promoted apoptosis rate of glioma cells (**Figure 8D**). Similarly, restoration of GPX7 following transfection with miR-29b mimic can reverse the miR-29b and erastin mediated synergistic inhibitory effects on glioma cells (**Figures 8A–D**). Apart from that, lip-1, fer-1 and DFO can abolish the increase of apoptosis rate of cells cotreated with miR-29b mimic and erastin (**Supplementary Figure S3C**). In summary, these results revealed that miR-29b can sensitize glioma cells to erastin partially *via* GPX7.

## Discussion

To date, some, but not all, molecules involved in glioma progression have been identified. In our study, multi-database analyses showed that GPX7 expression was upregulated in glioma and was an independent prognostic factor of glioma patients. GSEA revealed that some tumor-related signaling pathways and immunity-related activities are enriched in the GPX7 high expression group. Additionally, some immune cells were positively correlated with GPX7 expression in glioma.

Ferroptosis is a form of regulated cell death marked by lipid peroxidation (14). Previously, the ferroptosis inducers, for instance erastin, have shown potentials to eliminate malignancies, including glioma (36), melanoma (37) and other tumors (38). In addition, the combination of ferroptosis inducers and other therapies, such as chemotherapeutic agents and radiation, can result in stronger effects (36, 39). However, tumor cells can elevate the expression of other antioxidant genes that results in increased resistance to cell death (40). Yagoda N et al. reported that the expression of VDAC2/3, the targets of erastin on the outer mitochondrial membrane, markedly decreased after 10 h of erastin treatment, which led to erastin resistance (41). Elucidating more factors that regulate ferroptosis will certainly help to apply ferroptosis induction to anti-tumor therapies. In this study, we found GPX7 knockdown promoted lipid peroxidation and decreased the level of GSH. In addition, the combination of GPX7 deficiency and erastin treatment showed a remarkable synergistic effect on the induction of ferroptosis of glioma. Therefore, targeting GPX7 may help reverse the erastin resistance in glioma treatment.

As a peroxide sensor, GPX7 detoxifies peroxides and has been described to play essential roles in diseases. In normal oesophageal squamous epithelial cells, GPX7 knockdown can lead to the increase of intracellular ROS and oxidative DNA damage induced by pH4 bile acids, which increases the risk of oncogenesis (4). In glioma, our work found that GPX7 knockdown alone exerted no direct effect on tumor growth, although ferroptosis-related oxidative stress was promoted. We speculated that an external stimuli of oxidative stress, for instance erastin treatment, may be required for GPX7 targeting therapy of glioma.

Unlike other glutathione peroxidases, protein disulfide isomerase (PDI) and glucose-regulated protein GRP78, instead of GSH, are the main substrates of GPX7 (42, 43). GPX7 may exert antioxidant function through mechanisms different from GPX4, the key regulator of ferroptosis. In our study, we found that the loss of GPX7 resulted in decreased GSH level in glioma cells. This effect may be attributed to the activation of other antioxidant enzymes in the balance between the energy metabolism and oxidative damage resistance.

MiRNA-mRNA regulation has been identified as important regulatory mechanism in ferroptosis of tumors (15, 16, 23, 44). Based on algorithm prediction and experimental validation, our work found that miR-29b could directly inhibit GPX7 post-transcriptionally, exerting similar ferroptosis induction effect on glioma, synergizing with erastin treatment. In previous study, miR-29b was also found to promote oxidative stress in ischemic stroke (45). Therefore, miR-29b in oxidative stress is worthy of further study.

Some limitations were present: first, the survival data of patients in Huanhu hospital were missed. Second, we found GPX7 expression was associated with immune infiltration, but the specific roles of GPX7 in the immunomodulation are still unclear. Third, more investigations should be conducted into whether GPX7 affects the known ferroptosis-related signaling pathways and how GPX7 knockdown enhances the effects of erastin. Lastly, subcutaneous rather than intracranial *in situ* nude mice xenograft model was applied in our study due to the presence of the blood brain barrier which may prevent the penetration of erastin into the tumor. These possibly limit the cogency of our findings.

In summary, multi-center data revealed that GPX7 high expression was associated with poor clinical outcomes. GPX7 knockdown mediated enhancement of ferroptosis-related oxidative stress promoted glioma ferroptosis induced by erastin. Furthermore, miR-29b suppressed GPX7 expression post-transcriptionally in glioma. Reconstitution of miR-29b enhanced erastin sensitivity, partly *via* GPX7 suppression. All in all, suppressing GPX7 could be a valuable strategy for glioma treatment.

## Data Availability Statement

The original contributions presented in the study are included in the article/**Supplementary Material**. Further inquiries can be directed to the corresponding authors.

## Ethics Statement

The studies involving human participants were reviewed and approved by Ethics Committee of Huanhu Hospital of Tianjin Medical University. The patients/participants provided their written informed consent to participate in this study. The animal study was reviewed and approved by Animal Care and Used Committee of Tianjin Huanhu Hospital.

## Author Contributions

Study design: YZ, LX, and HY. Data collection: YZ, HW, and FW. Data analysis and interpretation: YZ, HW, and YY. Writing and review of the manuscript: YZ, XT, and HY. All authors contributed to the article and approved the submitted version.

## Funding

Our study was supported by National Natural Science Foundation of China (81972349), Tianjin Municipal Science and Technology Commission (20JCQNJC00410) and Tianjin Medical Key Discipline (Specialty) Construction Project.

## Conflict of Interest

The authors declare that the research was conducted in the absence of any commercial or financial relationships that could be construed as a potential conflict of interest.

## Publisher’s Note

All claims expressed in this article are solely those of the authors and do not necessarily represent those of their affiliated organizations, or those of the publisher, the editors and the reviewers. Any product that may be evaluated in this article, or claim that may be made by its manufacturer, is not guaranteed or endorsed by the publisher.

## Supplementary Material

Supplementary Figure S1 The expression of other GPX family members in glioma based on TCGA. **(A)** The expression differences of GPXs between normal and glioma were analyzed in GEPIA. **(B)** The multivariate Cox regression regarding GPXs in TCGA. **(C)** Some examples of high expression of GPX7 in patients with grade II and low expression in patients with grade III.

Supplementary Figure S2
**(A–D)** Bioinformatics analysis of the association of GPX7 and ferroptosis based on data from CGGA. **(A)** GSEA of ferroptosis and redox biology related gene sets in cohort with GPX7 high and low. **(B)** The different FPI levels among different WHO grades of glioma. **(C)** Kaplan-Meier analysis of OS according to the FPI level. **(D)** The association of GPX7 expression and FPI level in CGGA glioma samples. **(E)** CCK-8 assay was applied to analyze the viability of LN229 and T98G cells treated with different concentrations of RSL3 following the transfection with siGPX7 and siScr. **(F)** FITC annexin V and PI apoptosis assay was applied analyze the apoptosis rate of LN229 and T98G cells treated with RSL3 (1 μM) following the transfection with siGPX7 or siScr.

Supplementary Figure S3
**(A)** Immunofluorescence staining of LN229 and T98G cells subjected to siGPX7-ii or siScr transfection with or without erastin (10 μM) treatment for GPX7 (orange), Ki67 (green), MMP2 (red) and N-Cadherin (yellow). The nucleus is stained with DAPI (blue). Scale bar = 50 μm. **(B)** Apoptosis assay was used to analyze the apoptosis rate of cells treated with or without erastin (10 μM), fer-1 (2 μM), lip-1 (100 nM) and DFO (100 μM) following the transfection with siGPX7 and siScr. **(C)** Apoptosis assay was used to analyze the apoptosis rate of cells treated with or without erastin (10 μM), fer-1 (2 μM), lip-1 (100 nM) and DFO (100 μM) following the transfection with miR-29b mimic or NC mimic.
