# Supplementary figures and images for: GPX7 Is Targeted by miR-29b and GPX7 Knockdown Enhances Ferroptosis Induced by Erastin in Glioma
Source: Front Oncol. 2022 Jan 20;11:802124. doi: 10.3389/fonc.2021.802124 (PMC8811259; doi:10.3389/fonc.2021.802124)

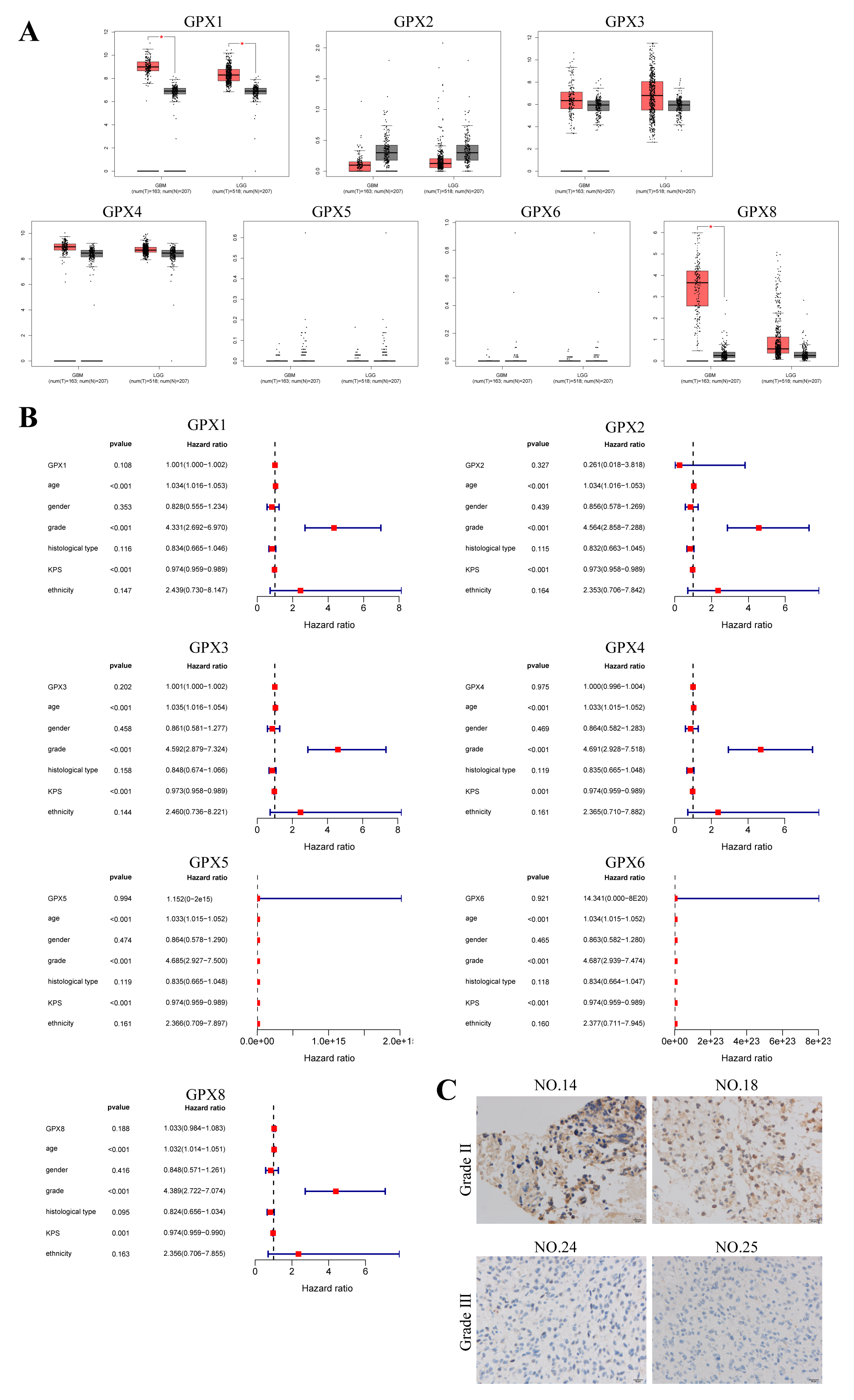

Supplement: Supplementary Figure S1 — The expression of other GPX family members in glioma based on TCGA. (A) The expression differences of GPXs between normal and glioma were analyzed in GEPIA. (B) The multivariate Cox regression regarding GPXs in TCGA. (C) Some examples of high expression of GPX7 in patients with grade II and low expression in patients with grade III. [file Image_1.tif]

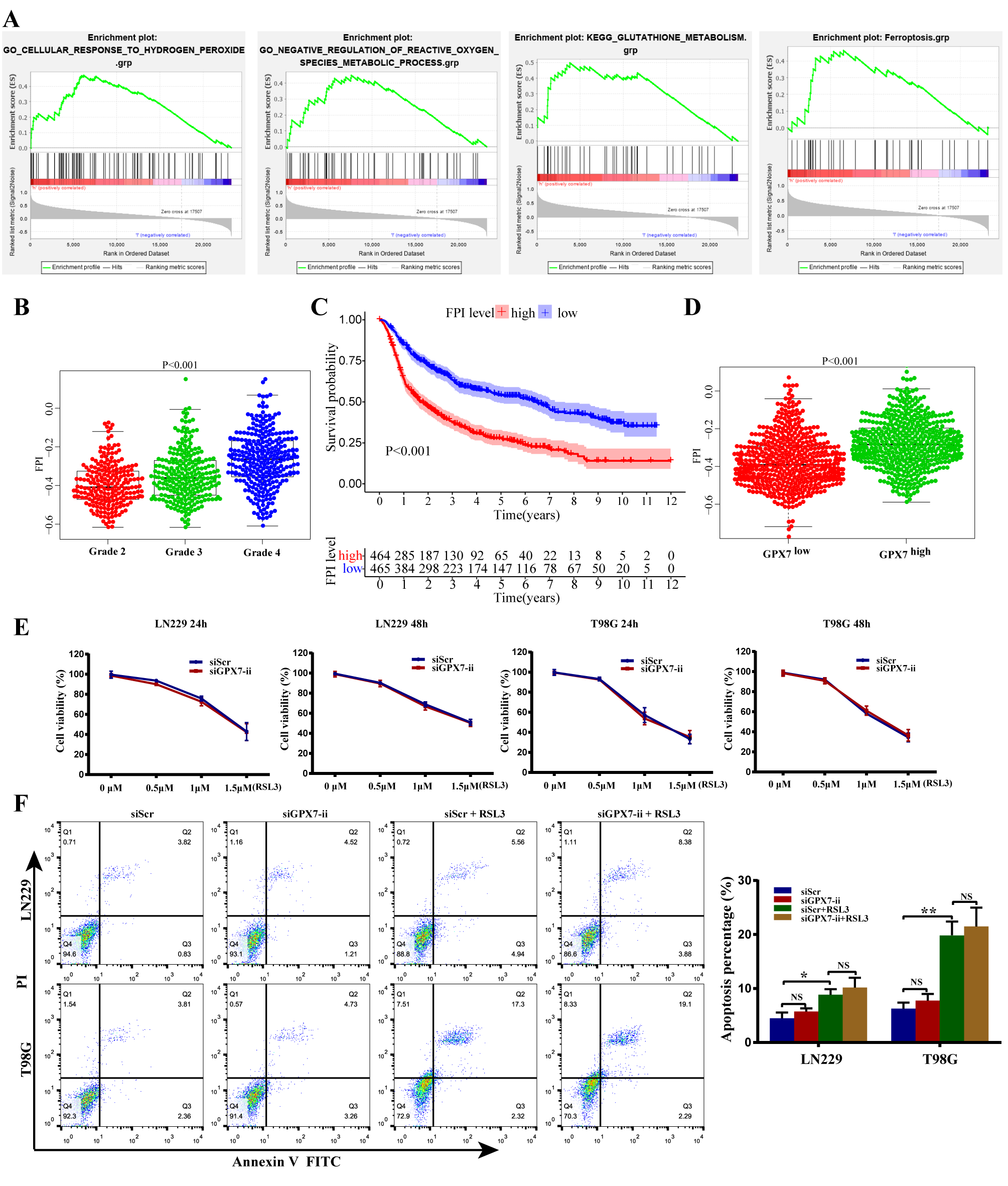

Supplement: Supplementary Figure S2 — (A–D) Bioinformatics analysis of the association of GPX7 and ferroptosis based on data from CGGA. (A) GSEA of ferroptosis and redox biology related gene sets in cohort with GPX7 high and low. (B) The different FPI levels among different WHO grades of glioma. (C) Kaplan-Meier analysis of OS according to the FPI level. (D) The association of GPX7 expression and FPI level in CGGA glioma samples. (E) CCK-8 assay was applied to analyze the viability of LN229 and T98G cells treated with different concentrations of RSL3 following the transfection with siGPX7 and siScr. (F) FITC annexin V and PI apoptosis assay was applied analyze the apoptosis rate of LN229 and T98G cells treated with RSL3 (1 μM) following the transfection with siGPX7 or siScr. [file Image_2.tif]

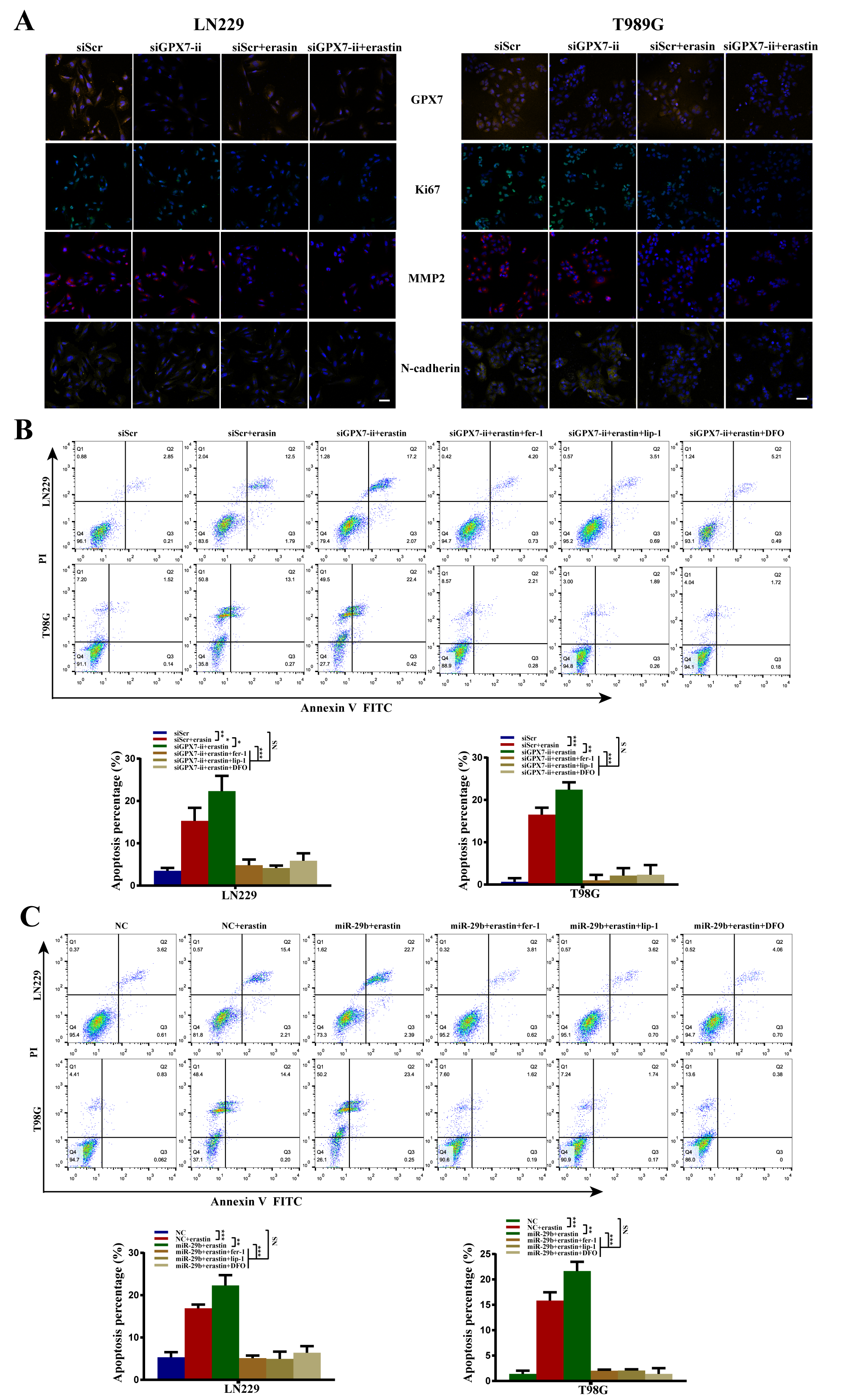

Supplement: Supplementary Figure S3 — (A) Immunofluorescence staining of LN229 and T98G cells subjected to siGPX7-ii or siScr transfection with or without erastin (10 μM) treatment for GPX7 (orange), Ki67 (green), MMP2 (red) and N-Cadherin (yellow). The nucleus is stained with DAPI (blue). Scale bar = 50 μm. (B) Apoptosis assay was used to analyze the apoptosis rate of cells treated with or without erastin (10 μM), fer-1 (2 μM), lip-1 (100 nM) and DFO (100 μM) following the transfection with siGPX7 and siScr. (C) Apoptosis assay was used to analyze the apoptosis rate of cells treated with or without erastin (10 μM), fer-1 (2 μM), lip-1 (100 nM) and DFO (100 μM) following the transfection with miR-29b mimic or NC mimic. [file Image_3.tif]
