# Supplementary material for: GPX7 Is Targeted by miR-29b and GPX7 Knockdown Enhances Ferroptosis Induced by Erastin in Glioma
Source: Front Oncol. 2022 Jan 20;11:802124. doi: 10.3389/fonc.2021.802124 (PMC8811259; doi:10.3389/fonc.2021.802124)
Supplement: Supplementary file 4 [file Table_1.docx]

**Table S1.** **The sense oligonucleotide sequences of siRNAs and miRNAs mimics**

|  | **Sequence** |
| --- | --- |
| siGPX7-i | GCACCUACAGUGUCUCAUUTT |
| siGPX7-ii, | GGAAGGAGCCCACCUGGAATT |
| scramble | UUCUCCGAACGUGUCACGUTT |
| miR-29a-3p | UAGCACCAUCUGAAAUCGGUUA |
| miR-29b-3p | UAGCACCAUUUGAAAUCAGUGUU |
| miR-29c-3p | UAGCACCAUUUGAAAUCGGUUA |
| LV3-shGPX7 | GGAAGGAGCCCACCTGGAA |

**Table S2. The details of patients’ characteristics in Huanhu dataset**

| **Number** | **Gender** | **Age**  **(year)** | **Histological diagnosis** | **Grade** | **IDH1 mutation** | **Ki67 index（%）** | **P53 mutation** | **MGMT methylation** | **1p19q codeletion** | **GPX7 expression** |
| --- | --- | --- | --- | --- | --- | --- | --- | --- | --- | --- |
| 1 | female | 31 | Astrocytoma | Ⅱ | Yes | 9.6 | Yes | Yes | NA | low |
| 2 | male | 31 | Astrocytoma | Ⅱ | Yes | 4 | Yes | Yes | No | high |
| 3 | female | 34 | Astrocytoma | Ⅱ | Yes | 2.6 | Yes | No | No | high |
| 4 | male | 54 | Astrocytoma | Ⅱ | Yes | 9.2 | Yes | Yes | No | low |
| 5 | male | 36 | Astrocytoma | Ⅱ | Yes | 6 | Yes | Yes | No | low |
| 6 | male | 39 | Astrocytoma | Ⅱ | Yes | 6.2 | Yes | Yes | NA | low |
| 7 | male | 41 | Astrocytoma | Ⅱ | Yes | 4 | Yes | Yes | No | low |
| 8 | male | 51 | Astrocytoma | Ⅱ | Yes | 2.5 | Yes | Yes | No | low |
| 9 | male | 35 | Astrocytoma | Ⅱ | Yes | 6.5 | Yes | Yes | No | low |
| 10 | male | 49 | Astrocytoma | Ⅱ | Yes | 4.1 | Yes | Yes | NA | low |
| 11 | female | 32 | Astrocytoma | Ⅱ | Yes | 6.8 | Yes | Yes | No | low |
| 12 | male | 54 | Astrocytoma | Ⅱ | Yes | 6.8 | Yes | Yes | No | low |
| 13 | female | 35 | Astrocytoma | Ⅱ | Yes | 3 | No | Yes | No | low |
| 14 | male | 51 | Astrocytoma | Ⅱ | Yes | 4.5 | Yes | Yes | No | high |
| 15 | female | 35 | Astrocytoma | Ⅱ | Yes | 7.8 | Yes | Yes | No | high |
| 16 | female | 38 | Astrocytoma | Ⅱ | Yes | 5.6 | Yes | No | NA | low |
| 17 | male | 36 | Astrocytoma | Ⅱ | Yes | 3.6 | Yes | Yes | NA | low |
| 18 | male | 45 | Astrocytoma | Ⅱ | Yes | 2.7 | Yes | No | NA | high |
| 19 | male | 45 | Astrocytoma | Ⅱ | Yes | 4 | Yes | NA | NA | low |
| 20 | female | 55 | Astrocytoma | Ⅲ | Yes | 29.2 | Yes | Yes | NA | high |
| 21 | male | 33 | Astrocytoma | Ⅲ | Yes | 29.2 | Yes | Yes | NA | high |
| 22 | male | 24 | Astrocytoma | Ⅲ | Yes | 13.2 | Yes | Yes | No | low |
| 23 | female | 51 | Astrocytoma | Ⅲ | Yes | 8 | Yes | Yes | No | high |
| 24 | male | 38 | Astrocytoma | Ⅲ | Yes | 17 | Yes | Yes | NA | low |
| 25 | male | 38 | Astrocytoma | Ⅲ | Yes | 30 | Yes | Yes | No | low |
| 26 | male | 36 | Astrocytoma | Ⅲ | Yes | 13.8 | Yes | Yes | NA | low |
| 27 | male | 45 | Astrocytoma | Ⅲ | Yes | 9.2 | Yes | Yes | No | low |
| 28 | male | 56 | Astrocytoma | Ⅲ | Yes | 7.5 | Yes | Yes | NA | low |
| 29 | female | 46 | GBM | Ⅳ | Yes | 42.5 | Yes | Yes | No | low |
| 30 | male | 55 | GBM | Ⅳ | Yes | 13.8 | Yes | Yes | No | low |
| 31 | male | 44 | GBM | Ⅳ | Yes | 30 | Yes | Yes | NA | low |
| 32 | female | 27 | GBM | Ⅳ | Yes | 20 | Yes | No | No | low |
| 33 | male | 43 | GBM | Ⅳ | Yes | 83 | Yes | Yes | No | high |
| 34 | female | 50 | GBM | Ⅳ | Yes | 32.6 | Yes | Yes | NA | high |
| 35 | male | 30 | GBM | Ⅳ | Yes | 45 | Yes | Yes | No | low |
| 36 | male | 37 | GBM | Ⅳ | Yes | 41.2 | Yes | Yes | NA | low |
| 37 | female | 40 | GBM | Ⅳ | Yes | 43.6 | Yes | Yes | NA | low |
| 38 | male | 36 | GBM | Ⅳ | Yes | 60 | Yes | Yes | No | high |
| 39 | female | 47 | GBM | Ⅳ | Yes | 25 | Yes | Yes | NA | high |
| 40 | female | 35 | GBM | Ⅳ | Yes | 70 | Yes | Yes | NA | low |
| 41 | female | 41 | GBM | Ⅳ | Yes | 27 | Yes | Yes | NA | low |
| 42 | male | 40 | Oligodendroglioma | Ⅱ | Yes | 8 | Yes | Yes | Yes | low |
| 43 | male | 48 | Oligodendroglioma | Ⅱ | Yes | 9.6 | Yes | Yes | Yes | low |
| 44 | female | 58 | Oligodendroglioma | Ⅱ | Yes | 9.6 | Yes | Yes | Yes | low |
| 45 | female | 39 | Oligodendroglioma | Ⅱ | Yes | 6 | Yes | Yes | Yes | high |
| 46 | male | 48 | Oligodendroglioma | Ⅱ | Yes | 12.8 | Yes | Yes | Yes | low |
| 47 | female | 47 | Oligodendroglioma | Ⅱ | Yes | 9.6 | Yes | Yes | Yes | low |
| 48 | female | 41 | Oligodendroglioma | Ⅱ | Yes | 9.2 | Yes | Yes | Yes | low |
| 49 | female | 50 | Oligodendroglioma | Ⅱ | Yes | 4 | Yes | Yes | Yes | low |
| 50 | male | 54 | Oligodendroglioma | Ⅱ | Yes | 9.2 | Yes | Yes | Yes | low |
| 51 | female | 29 | Oligodendroglioma | Ⅱ | Yes | 7 | No | Yes | Yes | low |
| 52 | male | 46 | Oligodendroglioma | Ⅱ | Yes | 8 | No | Yes | Yes | low |
| 53 | male | 36 | Oligodendroglioma | Ⅱ | Yes | 10 | Yes | Yes | Yes | low |
| 54 | female | 38 | Oligodendroglioma | Ⅱ | Yes | 9.2 | Yes | Yes | Yes | low |
| 55 | female | 21 | Oligodendroglioma | Ⅱ | Yes | 15 | Yes | Yes | Yes | low |
| 56 | male | 49 | Oligodendroglioma | Ⅱ | Yes | 7 | No | Yes | Yes | low |
| 57 | female | 61 | Oligodendroglioma | Ⅱ | Yes | 5 | Yes | Yes | Yes | low |
| 58 | male | 42 | Oligodendroglioma | Ⅱ | Yes | 5 | Yes | Yes | Yes | low |
| 59 | male | 40 | Oligodendroglioma | Ⅱ | Yes | 10 | Yes | Yes | Yes | low |
| 60 | male | 29 | Oligodendroglioma | Ⅱ | Yes | 4.8 | Yes | Yes | Yes | low |
| 61 | male | 45 | Oligodendroglioma | Ⅱ | Yes | 6.8 | Yes | Yes | Yes | low |
| 62 | male | 50 | Oligodendroglioma | Ⅲ | Yes | 15.8 | Yes | Yes | Yes | low |
| 63 | male | 63 | Oligodendroglioma | Ⅲ | Yes | 35.6 | Yes | Yes | Yes | high |
| 64 | male | 42 | Oligodendroglioma | Ⅲ | Yes | 10 | No | Yes | Yes | low |
| 65 | male | 33 | Oligodendroglioma | Ⅲ | Yes | 25 | Yes | Yes | Yes | low |
| 66 | female | 51 | Oligodendroglioma | Ⅲ | Yes | 40 | Yes | Yes | Yes | low |
| 67 | female | 36 | Oligodendroglioma | Ⅲ | Yes | 13.5 | Yes | Yes | Yes | low |
| 68 | male | 61 | Oligodendroglioma | Ⅲ | Yes | 19.8 | Yes | Yes | Yes | low |
| 69 | male | 43 | Oligodendroglioma | Ⅲ | Yes | 16 | Yes | Yes | Yes | low |
| 70 | female | 48 | Oligodendroglioma | Ⅲ | Yes | 13.2 | Yes | Yes | Yes | low |
| 71 | male | 58 | Oligodendroglioma | Ⅲ | Yes | 18 | Yes | Yes | Yes | low |
| 72 | male | 53 | Oligodendroglioma | Ⅲ | Yes | 15.2 | Yes | Yes | Yes | low |
| 73 | female | 55 | Oligodendroglioma | Ⅲ | Yes | 8 | Yes | Yes | Yes | low |
| 74 | female | 37 | Oligodendroglioma | Ⅲ | Yes | 19.8 | No | Yes | Yes | low |
| 75 | female | 39 | Oligodendroglioma | Ⅲ | Yes | 20 | Yes | Yes | Yes | low |
| 76 | female | 51 | Oligodendroglioma | Ⅲ | Yes | 42 | Yes | Yes | Yes | low |
| 77 | male | 57 | Oligodendroglioma | Ⅲ | Yes | 16.8 | Yes | Yes | Yes | low |
| 78 | male | 46 | Oligodendroglioma | Ⅲ | Yes | 19.6 | Yes | Yes | Yes | low |
| 79 | male | 55 | Oligodendroglioma | Ⅲ | Yes | 13.8 | Yes | Yes | Yes | low |
| 80 | male | 48 | Oligodendroglioma | Ⅲ | Yes | 22.8 | Yes | Yes | Yes | low |
| 81 | male | 47 | Oligodendroglioma | Ⅲ | Yes | 15.6 | Yes | Yes | Yes | low |
| 82 | female | 58 | Oligodendroglioma | Ⅲ | Yes | 13.2 | Yes | Yes | Yes | low |
| 83 | female | 40 | Oligodendroglioma | Ⅲ | Yes | 18.2 | Yes | Yes | Yes | low |
| 84 | male | 44 | Oligodendroglioma | Ⅲ | Yes | 32.8 | Yes | Yes | Yes | low |
| 85 | male | 53 | Oligodendroglioma | Ⅲ | Yes | 15.8 | Yes | Yes | Yes | low |
| 86 | female | 50 | Oligodendroglioma | Ⅲ | Yes | 16 | Yes | Yes | Yes | low |
| 87 | female | 35 | Oligodendroglioma | Ⅲ | Yes | 50 | Yes | Yes | Yes | low |
| 88 | female | 66 | GBM | Ⅳ | No | 32 | Yes | Yes | No | low |
| 89 | male | 60 | GBM | Ⅳ | No | 20 | Yes | No | No | high |
| 90 | male | 49 | GBM | Ⅳ | No | 26.2 | Yes | No | NA | low |
| 91 | female | 65 | GBM | Ⅳ | No | 35.2 | Yes | No | NA | low |
| 92 | female | 40 | GBM | Ⅳ | No | 34.2 | Yes | No | NA | low |
| 93 | female | 72 | GBM | Ⅳ | No | 38.2 | NA | NA | NA | high |
| 94 | male | 42 | GBM | Ⅳ | No | 40 | Yes | Yes | Yes | low |
| 95 | female | 40 | GBM | Ⅳ | No | 28.9 | Yes | No | NA | low |
| 96 | male | 61 | GBM | Ⅳ | No | 40 | Yes | No | No | low |
| 97 | male | 65 | GBM | Ⅳ | No | 32.8 | Yes | No | NA | low |
| 98 | male | 44 | GBM | Ⅳ | No | 28.9 | Yes | No | NA | high |
| 99 | male | 64 | GBM | Ⅳ | No | 35 | Yes | No | No | low |
| 100 | female | 62 | GBM | Ⅳ | No | 30 | Yes | Yes | NA | low |
| 101 | male | 48 | GBM | Ⅳ | No | 39.6 | Yes | No | NA | low |
| 102 | female | 67 | GBM | Ⅳ | No | 41.2 | Yes | No | NA | low |
| 103 | male | 55 | GBM | Ⅳ | No | 25 | Yes | Yes | NA | low |
| 104 | male | 54 | GBM | Ⅳ | No | 35.2 | Yes | No | NA | low |
| 105 | male | 64 | GBM | Ⅳ | No | 28.2 | Yes | Yes | NA | low |
| 106 | male | 61 | GBM | Ⅳ | No | 70 | Yes | Yes | NA | high |
| 107 | female | 82 | GBM | Ⅳ | No | 40 | Yes | Yes | NA | high |
| 108 | female | 41 | GBM | Ⅳ | No | 23.6 | Yes | No | NA | low |
| 109 | female | 60 | GBM | Ⅳ | No | 38 | Yes | No | No | low |
| 110 | male | 72 | GBM | Ⅳ | No | 60 | Yes | Yes | No | high |
| 111 | female | 39 | GBM | Ⅳ | No | 16.8 | NA | NA | NA | high |
| 112 | male | 50 | GBM | Ⅳ | No | 35.6 | Yes | Yes | NA | high |
| 113 | male | 60 | GBM | Ⅳ | No | 70 | Yes | No | NA | low |
| 114 | female | 67 | GBM | Ⅳ | No | 40 | Yes | No | NA | low |
| 115 | female | 67 | GBM | Ⅳ | No | 80 | Yes | Yes | NA | low |
| 116 | female | 59 | GBM | Ⅳ | No | 65 | Yes | Yes | NA | low |
| 117 | male | 39 | GBM | Ⅳ | No | 26.8 | Yes | No | NA | high |
| 118 | female | 59 | GBM | Ⅳ | No | 45 | Yes | Yes | Yes | high |
| 119 | male | 64 | GBM | Ⅳ | No | 15 | Yes | No | No | low |
| 120 | male | 52 | GBM | Ⅳ | No | 41.2 | Yes | Yes | NA | high |
| 121 | male | 59 | GBM | Ⅳ | No | 35.6 | Yes | No | NA | low |
| 122 | male | 51 | GBM | Ⅳ | No | 36.8 | Yes | Yes | NA | high |
| 123 | male | 65 | GBM | Ⅳ | No | 19.5 | Yes | Yes | NA | low |
| 124 | male | 17 | GBM | Ⅳ | No | 30 | Yes | Yes | NA | low |
| 125 | male | 66 | GBM | Ⅳ | No | 55 | Yes | Yes | NA | low |
| 126 | male | 68 | GBM | Ⅳ | No | 50 | Yes | No | NA | low |
| 127 | male | 69 | GBM | Ⅳ | No | 45 | Yes | No | NA | high |
